# Impact of chronic respiratory diseases on re-intubation rate in critically ill patients: a cohort study

**DOI:** 10.1038/s41598-021-88007-y

**Published:** 2021-04-21

**Authors:** Yanfei Shen, Weizhe Ru, Xinmei Huang, Shangzhong Chen, Jing Yan, Zhouxin Yang, Guolong Cai

**Affiliations:** 1 Department of Intensive Care, Zhejiang Hospital, No. 12, Lingyin Road, Hangzhou, 322100 Zhejiang People’s Republic of China; 2 Department of Oncology, Cixi People’s Hospital, Cixi, 315300 Zhejiang People’s Republic of China; 3 ENT Department, The Second Affiliated Hospital, Zhejiang University School of Medicine, Hangzhou, 310000 People’s Republic of China

**Keywords:** Medical research, Risk factors

## Abstract

Chronic respiratory diseases’ (CRDs) impact on re-intubation rate remains unclear. We investigated the association between these factors in mechanically ventilated patients. Data were extracted from the freely available online Medical Information Mart for Intensive Care III database. CRDs were defined according to ICD-9 codes. Generalised linear regression and propensity score matching were performed. Of 13,132 patients, 7.9% required re-intubation. Patients with chronic obstructive pulmonary disease (COPD) had higher re-intubation (OR 2.48, 95% CI 1.83–3.33) and mortality rates (OR 1.64, 95% CI 1.15–2.34) than those without. Patients with asthma had a lower mortality rate (OR 0.63, 95% CI 0.43–0.92) but a similar re-intubation rate to those of patients without. These findings remained stable after propensity score matching and bootstrapping analysis. The association of COPD with re-intubation was significantly stronger in patients with high oxygen-partial pressure (PaO_2_) or mild disease severity but was independent of carbon dioxide partial pressure. Corticosteroid use was associated with increased re-intubation rates in subgroups without CRDs (OR 1.77–1.99, p < 0.001) but not in subgroups with CRDs. COPD patients with high post-extubation PaO_2_ or mild disease severity should be carefully monitored as they have higher re-intubation and mortality rates.

## Introduction

Mechanical ventilation (MV) is a common respiratory support method in the intensive care unit (ICU). Millions of patients receive invasive MV in the United States each year^1,2^. Successful extubation is important for these patients, as extubation failure is associated with poor outcomes, including high mortality rates ranging from 25 to 50%^3–5^. However, despite multiple weaning protocols, such as the spontaneous breathing trial^6^, the overall re-intubation rate remains high at 10%^3,7^, and even exceeds 20% in high-risk patients^8^. Thus, identifying patients with a high re-intubation risk may be important for clinicians.

Chronic respiratory diseases (CRDs) such as chronic obstructive pulmonary disease (COPD), asthma, and bronchiectasis are common comorbidities in patients in ICUs. Impaired respiratory strength is common in patients with CRDs and often leads to poor efficiency in ventilation^9–11^, even in patients who are stable. Evidence indicates that altered inspiratory and expiratory strength often leads to an increased risk of extubation failure^12,13^, and studies have reported re-intubation rates as high as 20–30% in patients with CRDs^14–16^.

One cohort study^5^ has reported that chronic cardiac or respiratory disease may be a risk factor for extubation failure in patients aged > 65 years. However, the heterogeneous effect of different CRDs on re-intubation rate among ICU patients on MV has not been well established.

This study aimed to investigate the associations between different CRDs and re-intubation rate and evaluate the impact of corticosteroids on re-intubation rate. We hypothesised that different CRDs may have differing effects on the prognosis of patients in ICUs.

## Results

### Baseline characteristics

A total of 13,132 patients were included in this analysis (Supplementary Fig. S1). The re-intubation rate was 7.9% (Table 1). The duration of the first intubation and the total ICU length of stay were significantly longer in the re-intubation group than in the successful extubation group. Overall, the proportion of patients with CRDs was 9.5% (1250/13,132) in this cohort. Compared to that in the successful extubation group, the proportion of patients with COPD was significantly higher in the re-intubation group (61/1042 vs. 255/12,090, p < 0.001), whereas the proportion of patients with asthma was comparable in both groups (75/1042 vs. 800/12,090, p = 0.471). Patients in the re-intubation group were more likely to receive corticosteroids after extubation. The detailed comparisons of baseline characteristics are presented in Table 1.

**Table 1:** Baseline comparisons between successful extubation and re-intubation groups.

Demographics	Successful extubation (n = 12,090)	Re-intubation (n = 1042)	All patients (n = 13,132)	p
Age (years)	64.4 ± 15.6	65.8 ± 15.4	64.5 ± 15.6	0.004
Male [n (%)]	7379 (61.0)	585 (56.1)	7982 (60.8)	0.001
Weight (kg)	83.2 ± 22.2	82.0 ± 24.5	83.1 ± 22.4	0.113
**Laboratory indexes**				
Initial white blood cell count (10^9^/L)	12.2 ± 4.4	12.9 ± 4.9	12.3 ± 4.4	< 0.001
Initial haemoglobin level (g/dL)	10.4 ± 1.3	10.2 ± 1.3	10.3 ± 1.3	< 0.001
Initial platelet count (10^9^/L)	201.3 ± 104.9	222.3 ± 131.7	202.9 ± 107.4	< 0.001
Initial serum creatinine (mg/dL)	1.1 ± 0.9	1.3 ± 1.0	1.1 ± 0.9	< 0.001
Initial serum sodium (mmol/L)	138.9 ± 3.6	140.1 ± 4.1	138.9 ± 3.6	< 0.001
SOFA at ICU admission [median (IQR)]	5 (3–7)	6 (4–8)	5 (3–7)	< 0.001
Maximum SOFA [median (IQR)]	9 (7–11)	11 (9–14)	9 (7–11)	< 0.001
**ICU types**				< 0.001
MICU	2643 (21.8)	359 (34.4)	3002 (22.8)	
CCU	6912 (57.1)	377 (36.1)	7289 (55.5)	
SICU/TICU	2535 (20.9)	306 (29.3)	2841 (21.6)	
**Comorbidities**				
Hypertension [n (%)]	5667 (46.8)	383 (36.7)	6050 (46.0)	< 0.001
Diabetes mellitus [n (%)]	3083 (25.5)	245 (23.5)	3328 (25.3)	0.157
Coronary diseases [n (%)]	4811 (39.8)	281 (26.9)	5092 (38.8)	< 0.001
COPD [n (%)]	255 (2.1)	61 (5.8)	316 (2.4)	< 0.001
Asthma [n (%)]	800 (6.6)	75 (7.2)	875 (6.7)	0.471
Bronchiectasis [n (%)]	33 (0.3)	6 (0.6)	39 (0.3)	0.085
Chronic respiratory diseases [n (%)]	1107 (9.2)	143 (13.7)	1250 (9.5)	< 0.001
Sepsis [n (%)]	3416 (28.2)	705 (67.6)	4121 (31.3)	< 0.001
**Hemodynamic indexes**				
Fluid balance > 0 [n (%)]	8225 (68.0)	785 (75.3)	9010 (68.6)	< 0.001
Vasopressor-use within 24 h [n (%)]	2740 (22.7)	330 (31.6)	3070 (23.3)	< 0.001
Post-extubation corticosteroids use	1387 (11.5)	234 (22.4)	1621 (12.3)	< 0.001
**Clinical outcomes**				
Duration of first intubation (hours)	41.9 ± 74.5	103.5 ± 159.1	46.8 ± 86.6	< 0.001
ICU length of stay [days, median (IQR)]	2.4 (1.3–4.6)	4.2 (2.0–9.0)	2.5 (1.3–4.8)	< 0.001
Hospital length of stay [days, median (IQR)]	8.3 (4.3–13.1)	18.7 (12.4–27.6)	8.9 (6–14.4)	< 0.001
ICU mortality [n (%)]	233 (1.9)	188 (18.0)	421 (3.2)	< 0.001
In-hospital mortality [n (%)]	493 (4.1)	225 (21.6)	718 (5.5)	< 0.001

*SOFA* sequential organ failure assessment, *MICU* medical intensive care unit, *CCU* including coronary care unit and post cardiac surgery care unit, *SICU* surgical intensive care unit, *TICU* traumatic intensive care unit, *COPD* chronic obstructive pulmonary disease, *ICU* intensive care unit.

### Re-intubation rate within different CRDs

#### Crude comparisons

The crude re-intubation rate and the duration of the first intubation were significantly lower in patients without CRDs than in those with CRDs (899/11,882 patients vs. 143/1250 patients, p < 0.001, and 14 [range 5–44] hours vs. 17 [range 6–61] hours, p < 0.001, respectively; Table 2). Likewise, in the comparison between patients without and with COPD, the re-intubation rate (981/11,835 [8.2%] vs. 61/316 [19.3%], respectively; p < 0.001) and the duration of the first intubation (14 [range 5–44] hours vs. 29 [range 9–78] hours, respectively; p < 0.001) were significantly lower in patients without COPD. However, the re-intubation rate and the duration of the first intubation were not significantly different between the asthma and no-asthma groups.

**Table 2:** Crude comparisons of clinical outcomes within different subgroups.

Clinical outcomes	No CRDs (n = 11,882)	CRDs (n = 1250)	p	No COPD (n = 11,835)	COPD (n = 316)	p	No asthma (n = 12,257)	Asthma (n = 875)	p
Re-intubation rate [n (%)]	899 (7.6)	143 (11.4)	< 0.001	981 (8.2)	61 (19.3)	< 0.001	967 (7.9)	75 (8.7)	0.471
Duration of first intubation (h)	14 (5–44)	17 (6–61)	< 0.001	14 (5–44)	29 (9–78)	< 0.001	15 (5–45)	15 (5–56)	0.091
ICU length of stay [median (IQR)]	3.1 (1.8–6.0)	3.3 (2.0–6.4)	< 0.001	3.1 (1.8–6.0)	4.5 (2.5–8.2)	< 0.001	3.1 (1.8–6.0)	3.1 (1.8–6.0)	0.833
Hospital length of stay [median (IQR)]	8.8 (6.0–14.4)	9.1 (5.9–14.4)	0.821	6.0 (4.3–8.9)	9.7 (5.9–15.7)	0.191	8.9 (6–14.5)	8.8 (5.9–13.6)	0.214
In-hospital mortality [n (%)]	642 (5.4)	76 (6.1)	0.317	678 (5.7)	40 (12.6)	< 0.001	688 (5.6)	30 (3.4)	0.006

*CRDs* chronic respiratory diseases, *COPD* chronic obstructive pulmonary disease, *ICU* intensive care unit, *IQR* inter quartile range.

#### Adjusted outcomes

Potential confounders were adjusted in the multivariable logistic models (Table 3). The odds ratio (OR) for re-intubation was significant in patients with CRDs (OR 1.51, 95% CI 1.24–1.83, p < 0.001) and in those with COPD (OR 2.48, 95% CI 1.83–3.33, p < 0.001). However, for asthma, the OR for re-intubation was non-significant. All results were found to be stable in the logistic models after applying bootstrap techniques with 1000 resamples.

**Table 3:** Associations between clinical outcomes and different respiratory diseases.

	CRDs			COPD			Asthma		
	Multivariable logistic model, aOR (95% CI)^a^	p	Multivariable logistic model with bootstrapping, aOR (95% CI)^b^	Multivariable logistic model, aOR (95% CI)^a^	p	Multivariable logistic model with bootstrapping, aOR (95% CI)^b^	Multivariable logistic model, aOR (95% CI)^a^	p	Multivariable logistic model with bootstrapping, aOR (95% CI)^b^
Re-intubation	1.51 (1.24–1.83)	< 0.001	1.50 (1.24–1.81)	2.48 (1.83–3.33)	< 0.001	2.47 (1.76–3.46)	1.09 (0.85–1.41)	0.486	1.10 (0.82–1.44)
In-hospital mortality	1.02 (0.78–1.31)	0.901	1.01 (0.79–1.29)	1.64 (1.15–2.34)	0.007	1.64 (1.13–2.37)	0.63 (0.43–0.92)	0.019	0.62 (0.40–0.97)

*CRDs* chronic respiratory diseases, *COPD* chronic obstructive pulmonary disease, *aOR* adjusted odds ratio.

^a^Multivariable logistic model was used to evaluate the association between clinical outcomes and different respiratory diseases. All these models were adjusted for same co-variables, including age, hypertension, coronary disease, renal replacement therapy, duration of intubation, haemoglobin level, white blood cell count, serum sodium level, and vasopressor use.

^b^95% CI using bootstrap techniques (100 resamples) was adjusted for the same co-variables listed above.

#### Outcomes after PSM

The comparison of re-intubation rate was also verified using PSM. A total of 316 patients from the COPD group and 1147 patients from the no-COPD group were well matched using a 1:4 matching algorithm (Table 4). The overall quality of the matched sample was assessed by graphically inspecting the propensity scores between the groups (Supplementary Fig. S2), and the mean standardised difference was < 10% in all covariates, which demonstrated substantial improvement in covariate balance across the treatment groups. Among the matched cases, re-intubation was significantly higher in the COPD group (61/316 vs. 90/1147, p < 0.001), and patients with COPD were more likely to have a longer intubation duration and ICU stay. However, in matched patients with and without asthma, the re-intubation rates were comparable between these two groups.

**Table 4:** Comparisons of clinical outcomes after propensity score matching.

Variables	No COPD (n = 1147)	COPD (n = 316)	p	No asthma (n = 2865)	Asthma (n = 875)	p
Re-intubation rate [n (%)]	90 (12.8)	61 (19.3)	< 0.001	237 (8.1)	75 (8.5)	0.779
In-hospital mortality [n (%)]	89 (7.3)	40 (12.6)	0.007	156 (4.9)	30 (3.4)	0.016
Duration of first intubation (hours)	15 (5–57)	29 (9–78)	< 0.001	15 (5–48)	15 (5–56)	0.411
ICU length of stay [median (IQR)]	3.3 (1.9–6.5)	4.5 (2.5–8.2)	< 0.001	3.1 (1.8–6.2)	3.1 (1.8–6.0)	0.459
Hospital length of stay [median (IQR)]	8.9 (6.1–14.1)	9.7 (5.9–15.7)	0.312	8.7 (6.0–14.2)	11.1 (5.5–16.5)	0.260

The following variables were selected to generate the propensity score: age, sex, hypertension, diabetes, renal replacement therapy, maximum sequential organ failure assessment score during ICU stay, duration of intubation, any vasopressor use within the first 24 h, serum creatinine level, haemoglobin level, potassium level, and sodium level. Gamma for COPD 2.1, p 0.063.

*COPD* chronic obstructive pulmonary disease, *ICU* intensive care unit, *IQR* inter quartile range.

#### Interaction between COPD and clinical parameters

**Figure 1:**
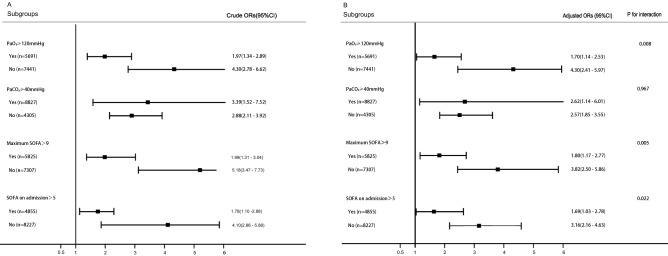
Factors influencing the impact of COPD on re-intubation rate. The impact of COPD on re-intubation rate was found to be affected by post-extubation PaO_2_ and SOFA but not by PaCO_2_. *SOFA* sequential organ failure assessment, *COPD* chronic obstructive pulmonary disease, *PaCO*_*2*_ carbon dioxide partial pressure.

A significant interaction was detected between COPD and post-extubation oxygen partial pressure (PaO_2_, p = 0.008; Fig. 1). The association between COPD and re-intubation rate was considerably stronger in patients with high PaO_2_ (≥ 120 mmHg, OR 4.30, 95% CI 2.41–5.97, p < 0.001) than in those with low PaO_2_ (< 120 mmHg, OR 1.70, 95% CI 1.14–2.53, p = 0.009). A significant interaction was also found between COPD and Sequential Organ Failure Assessment (SOFA) scores (both the initial and maximum scores), and the association between COPD and the re-intubation rate was considerably stronger in patients with mild disease severity, than the association in those with severe disease severity. No significant interaction was found between COPD and carbon dioxide partial pressure (PaCO_2_) in the current study.

#### Impact of corticosteroids on re-intubation

**Figure 2:**
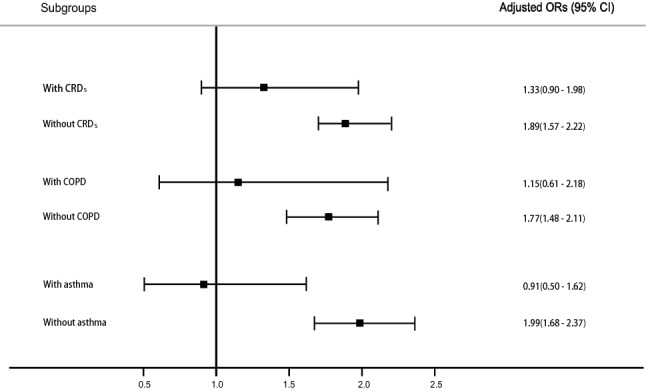
Association between post-extubation corticosteroid use and re-intubation rate in patients with different respiratory diseases. The p for interaction is 0.199, 0.155, and 0.029 in groups with/without CRDs, COPD, and asthma, respectively. *COPD* chronic obstructive pulmonary disease, *CRDs* chronic respiratory diseases.

The association between post-extubation corticosteroid use and re-intubation rate was investigated in different subgroups. After adjusting for covariates, corticosteroid use was found to be consistently associated with an increased re-intubation rate in the subgroups without respiratory diseases (ORs 1.77–1.99, p < 0.001; Fig. 2), but its association was non-significant in the subgroups with respiratory diseases (CRDs: OR 1.33, p = 0.142; COPD: OR 1.15, p = 0.660; asthma: OR 0.9, p = 0.748).

### In-hospital mortality rates in terms of different CRDs

#### Crude comparisons

The crude in-hospital mortality rate was higher in the COPD group than in the no-COPD group (40/316 vs. 678/11,835, p < 0.001; Table 2), while the in-hospital mortality rate was lower in the asthma group than in the no-asthma group (30/875 vs. 688/12,257, respectively; p = 0.006).

#### Adjusted outcomes

After adjusting for confounders in the multivariable logistic models (Table 3), COPD was associated with an increased risk of in-hospital mortality (OR 1.64, 95% CI 1.15–2.34, p = 0.007). However, asthma appeared to be a protective factor for in-hospital mortality (OR 0.63, 95% CI 0.43–0.92, p = 0.019). The overall OR for in-hospital mortality concerning CRDs was not significant.

#### Outcomes after PSM

Among matched patients with and without COPD, the in-hospital mortality rate was significantly higher in the COPD patient group (40/316 vs. 89/1147, p = 0.007). In matched patients with and without asthma, a decreased in-hospital mortality rate was found in the asthma group (30/875 vs. 156/2865, p = 0.016).

## Discussion

There were three major findings in this study. First, CRDs were present in 9.5% of critically ill patients on MV, and different CRDs had different impacts on the prognosis of these patients. Second, the re-intubation rate was significantly higher in patients with COPD, and this association was even stronger in patients with mild disease severity or high post-extubation PaO_2_, but was independent of post-extubation PaCO_2_. In patients with asthma, the association with re-intubation rate was non-significant. Third, post-extubation corticosteroid use was significantly associated with an increased re-intubation rate in patients without CRDs, but was non-significant in patients with CRDs. These findings remained stable after adjusting for covariates and PSM.

Successful extubation plays an essential role in critical care as both prolonged MV and extubation failure have been found to be associated with a poor prognosis^3,17^. However, current re-intubation rates remain as high as 10–20%. Strategies to identify patients at high risk for extubation failure are essential to improve the management of weaning and extubation. Many risk factors, such as age, disease severity, and intubation duration, have been reported for extubation failure^18^. However, studies assessing the effect of different CRDs on the re-intubation rate remain scarce.

One cohort study^5^ investigated the extubation failure rate in medical ICU patients and found that mixed chronic cardiac or respiratory disease may be a risk factor for extubation failure in patients aged > 65 years (34% vs. 9%, p < 0.01). However, owing to that study’s limited sample size, chronic respiratory and cardiac diseases were classified as one group and the adjusted impact of different CRDs was not investigated. Chu et al.^19^ assessed factors associated with re-intubation within 14 days after weaning from the ventilator and found that the primary reasons for intubation, including COPD, were also significant risks for re-intubation. Different from our study, the overall re-intubation rate was significantly low in Chu et al.’s study (2.8% vs 7.9%), suggesting possible heterogeneity between the two cohorts. In addition, only acute exacerbation of COPD was analysed and the impact of stable COPD or other CRDs remained undetermined.

In this study, we found that different CRDs had different impacts on re-intubation rate. A significant association between COPD and re-intubation rate was confirmed in the multivariable logistic model and PSM. This finding was not unexpected, as a prolonged high workload of the respiratory muscles makes respiratory muscle fatigue common in these patients^20–22^. Moreover, we noted that this association interacted with PaO_2_ but was independent of PaCO_2_. The possible mechanisms cannot be inferred due to the retrospective design of our study. However, several studies have indicated that in patients with COPD receiving oxygen therapy^23^, the respiratory response to CO_2_ could be blunted due to high PaO_2_, which leads to hypoventilation after extubation. Furthermore, it has been reported that hyperoxia is associated with lung oxidant/antioxidant imbalance^24,25^. Whether these mechanisms contribute to an increased re-intubation risk in patients with high PaO_2_ requires further investigation.

In clinical practice, an elevated but stable PaCO_2_ is often permitted in patients with COPD requiring extubation. However, whether this mild hypercapnia may lead to a higher extubation failure in patients with COPD has not been investigated. Contou et al.^26^ reported that in patients with severe cardiogenic pulmonary oedema, hypercapnia did not increase the rate of intubation or prolong non-invasive ventilation. However, no confounder correction or interaction analysis was performed due to the small sample size. In this study, we found the association between COPD and re-intubation rate was not affected by post-extubation PaCO_2_, after adjusting for confounders. However, we also noted that 95% of the patients in our study had a post-extubation PaCO_2_ of < 55 mmHg. Therefore, whether a higher PaCO_2_ would have resulted in a different interaction with COPD should be investigated in future studies.

Furthermore, we found that the association between COPD and re-intubation rate was even stronger in the patient subgroup with mild disease severity, than the association in those with severe disease severity. In patients with severe disease severity, many factors may lead to extubation failure, such as low PaO_2_/FiO_2_^27^, a rapid shallow breathing index^28^, and ICU-related weakness and respiratory muscle insufficiency^12,13,29–31^. Therefore, it is possible that the association between COPD and re-intubation may be weakened or even undetectable in patients with severe condition.

We also noted that the association between asthma and re-intubation was not significant. In contrast to patients with COPD, patients with asthma may have had relatively normal respiratory function, which could explain this result. We also observed that asthma was significantly associated with decreased mortality. This finding has not previously been reported concerning patients in ICUs. However, similar findings have been reported in patients with influenza. In a multicentre retrospective analysis involving 1520 patients with influenza, Myles et al.^32^ found that patients with asthma were less likely to require intensive care or die compared with those without asthma. Similarly, two other studies^33,34^ have also reported that patients with a history of asthma had a lower in-hospital mortality rate than that of patients without asthma. Several reasons have been proposed for these findings^32–34^, such as early admission, mild disease severity, and rapid response to treatment. In our study, we noted that patients with asthma were significantly younger that those without asthma. However, our findings remained stable after adjusting for this factor. More studies are required to further investigate possible relevant mechanisms.

Finally, we found that corticosteroid use was associated with an increased re-intubation rate in subgroups without CRDs, but not in subgroups with CRDs. Concerning patients post-extubation, corticosteroids are more commonly prescribed for patients with extubation failure risk factors, such as hypoxemia and lung infiltration. Therefore, there is a risk that the true efficacy of corticosteroid use may be offset by its close relationship with extubation failure factors, due to its selective use in clinical practice^35^. However, this finding still indicates that the efficacy of corticosteroids may vary in relation to CRDs, and this needs to be further investigated in future studies.

This study had several limitations. First, the MIMIC III recorded data from 2001 to 2012, during which time there were major changes to extubation guidelines. Therapeutic bias should be considered when interpreting the findings. Second, due to our study’s retrospective design, specific reasons for ICU admission or MV could not be ascertained and, therefore, were not included in the analysis. Moreover, although we included as many potential confounders as possible, there remains a risk that some potential confounding factors, such as non-invasive ventilation application, COPD severity, or pulmonary function status, were not accounted for. Third, only COPD and asthma were analysed in the current study, and due to the limited number of patients, other types of CRDs such as bronchiectasis were not evaluated. Furthermore, re-intubation was defined as all re-intubation events occurring during the entire hospital stay. Whether our findings would vary if we used different re-intubation definitions remains unclear. Finally, this was a single-centre study, and the applicability of the conclusions remains uncertain. Nevertheless, our study findings may provide a solid foundation for further research.

In conclusion, this study showed that different CRDs may impact patients on MV differently. The association between COPD and re-intubation rate was influenced by disease severity and PaO_2_. More attention should be paid to patients with COPD after extubation, especially to those with mild disease severity. Titrating oxygen saturation to avoid hyperoxia may be beneficial. More studies are required to investigate the underlying mechanisms in relation to our observations and to further validate our findings.

## Methods

### Data sources

Data were extracted from the Medical Information Mart for Intensive Care III (MIMIC III)^36^ database, which is an online database containing detailed information concerning > 40,000 patients in ICUs of Beth Israel Deaconess Medical Centre. Patient information was anonymised for privacy. The Institutional Review Board of Massachusetts Institute of Technology for Publications approved publication of the database information and of this study and waived the requirement for informed patient consent. Researchers who passed an online training test and were authorized to access this database. Y.S. was responsible for data extraction (certification NO. 1564657). The study protocol was performed in accordance with the relevant guidelines.

### Patient selection

All patients who had intubation records were initially screened, and those with the following conditions were excluded: records indicating tracheotomy status, no record of extubation, age < 18 years, length of ICU stay < 24 h, and a diagnosis indicating intracranial haemorrhage. If one patient was found to have had multiple admissions or re-intubation records, only data from their first admission or first re-intubation were used.

### Data extraction

Data concerning patient demographics and comorbidities were extracted. As the current study aimed to determine the potential risk for re-intubation, other information such as biochemical and drug records was also recorded within the period between extubation and re-intubation. Re-intubation was the primary endpoint, which was defined as all re-intubation events that occurred throughout a hospital stay. In-hospital mortality was the secondary endpoint.

### Definition of CRDs

COPD, asthma, bronchiectasis, and pulmonary or pleural tuberculosis were defined as CRDs. Three diagnostic codes indicating COPD were identified in the MIMIC III database. Diagnostic codes referring to any form of asthma (for example, extrinsic asthma, intrinsic asthma, acute asthma exacerbation, cough variant asthma) were considered as indicative of asthma. The detailed ICD-9 codes for these diseases are listed in the Supplementary file 1.

### Definition of corticosteroid use

Details on the systemic use (enteral or intravenous) of different types of corticosteroids (for example, dexamethasone, methylprednisolone, prednisone, hydrocortisone) were extracted from this database. Inhalatory corticosteroid use was excluded. Patients with records indicating corticosteroid use within the extubation and re-intubation periods were designated as a corticosteroid use group.

### Missing data management

Most continuous variables in the current study had < 5% missing values, which were replaced with their mean or median values. Variables with > 20% missing values were not filled. For dichotomous variables, a missing value was replaced with a default value (zero).

### Statistical analysis

Continuous variables were presented as mean (± standard deviation) or median (interquartile range). A student’s t-test or Wilcoxon rank-sum test was used as appropriate. Categorical data were compared using a chi-square test and expressed as proportions. To adjust for the potential impact of possible confounders, variables with a p-value < 0.2 in the univariable comparison were included in the initial model. A stepwise approach method was used to build the final logistic model. Multicollinearity was tested using the variance inflation factor (VIF) method, with a VIF ≥ 5 indicating multicollinearity. To test the stability of these logistic models, bootstrap techniques using 1000 resamples were performed. Subgroup analysis was performed in patients with COPD or asthma. Propensity score matching (PSM) was used to minimise the effect of confounding factors such as biochemical indices and disease severity, which could have led to biased outcomes. The propensity score was estimated using a logistic regression model. A one-to-four nearest-neighbour-matching algorithm was applied with a calliper of 0.05. Age, sex, hypertension, diabetes, renal replacement therapy, maximum SOFA score during ICU stay, duration of intubation, vasopressor use within the first 24 h, serum creatinine level, haemoglobin level, potassium level, and sodium level were selected to generate the propensity score. A bias in the mean (or proportion) of the covariates between the two groups was examined using the standardised difference before and after PSM^37,38^. To test for bias due to imbalance in unmeasured covariates in the PSM, sensitivity analyses were performed to quantify the degree of hidden bias needed to invalidate the main conclusions. A two-tailed test was performed, and a p-value < 0.05 was considered statistically significant. All statistical analyses were performed using STATA version 14.0 (College Station, TX, USA) software.

## Supplementary Information

Supplementary Information.
