# Supplementary material for: Impact of chronic respiratory diseases on re-intubation rate in critically ill patients: a cohort study
Source: Sci Rep. 2021 Apr 21;11:8663. doi: 10.1038/s41598-021-88007-y (PMC8060362; doi:10.1038/s41598-021-88007-y)
Supplement: Supplementary file 1 — Supplementary Information. [file 41598_2021_88007_MOESM1_ESM.docx]

**Title page**

**TITLE:** Impact of chronic respiratory diseases on re-intubation rate in critically ill patients: A cohort study

Yanfei Shen, MM^1^; Weizhe Ru, MM^2*^; Xinmei Huang, MM^3*^; Guolong Cai, MM^1*^; Shangzhong Chen, MM^1^; Jing Yan, MD^1^; Zhouxin Yang, MD^1^

^1^ Department of Intensive Care, Zhejiang Hospital, Hangzhou, Zhejiang, P.R. China.

^2^ Department of Oncology, Cixi People's Hospital, Cixi, Zhejiang, 315300, P.R. China.

^3^ Department of Internal Medicine, Zheda Hospital of Zhejiang University, 38#, Zheda Road, Hangzhou, 310000, P.R. China

* Weizhe Ru, Xinmei Huang, and Guolong Cai are co-first authors

**Corresponding author:**

Zhouxin Yang, MD

Department of Intensive Care, Zhejiang Hospital, No. 12, Lingyin Road, Hangzhou, Zhejiang, 322100, P.R. China. Phone: +86--138-0579-0809, Fax: 86-0579-86856041

E-mail: yangzhouxing1979@126.com

**SQL code used for extraction in MIMIC III database**

**Code for asthma**

select * from mimiciii.diagnoses_icd where icd9_code in (select icd9_code from mimiciii.d_icd_diagnoses where lower(long_title) like '%asthma%' and not (icd9_code='9757' or icd9_code='E9457' or icd9_code='V175'))

**Code for bronchiectasis**

select * from mimiciii.diagnoses_icd where icd9_code in (select icd9_code from mimiciii.d_icd_diagnoses where lower(long_title) like '%bronchie%')

**Code for pulmonary or pleural tuberculosis**

select * from mimiciii.diagnoses_icd where icd9_code in (select icd9_code from mimiciii.d_icd_diagnoses where lower(long_title) like '%tuber%' and (lower(long_title) like '%pneumo%' or lower(long_title) like '%pulmo%' or lower(long_title) like '%pleur%' or lower(long_title) like '%trache%' or lower(long_title) like '%respira%' or lower(long_title) like '%lung%' or lower(long_title) like '%bronch%'))

**Code for COPD**

select * from mimiciii.d_icd_diagnoses where icd9_code= '49120' or icd9_code= '49121' or icd9_code= '49122'


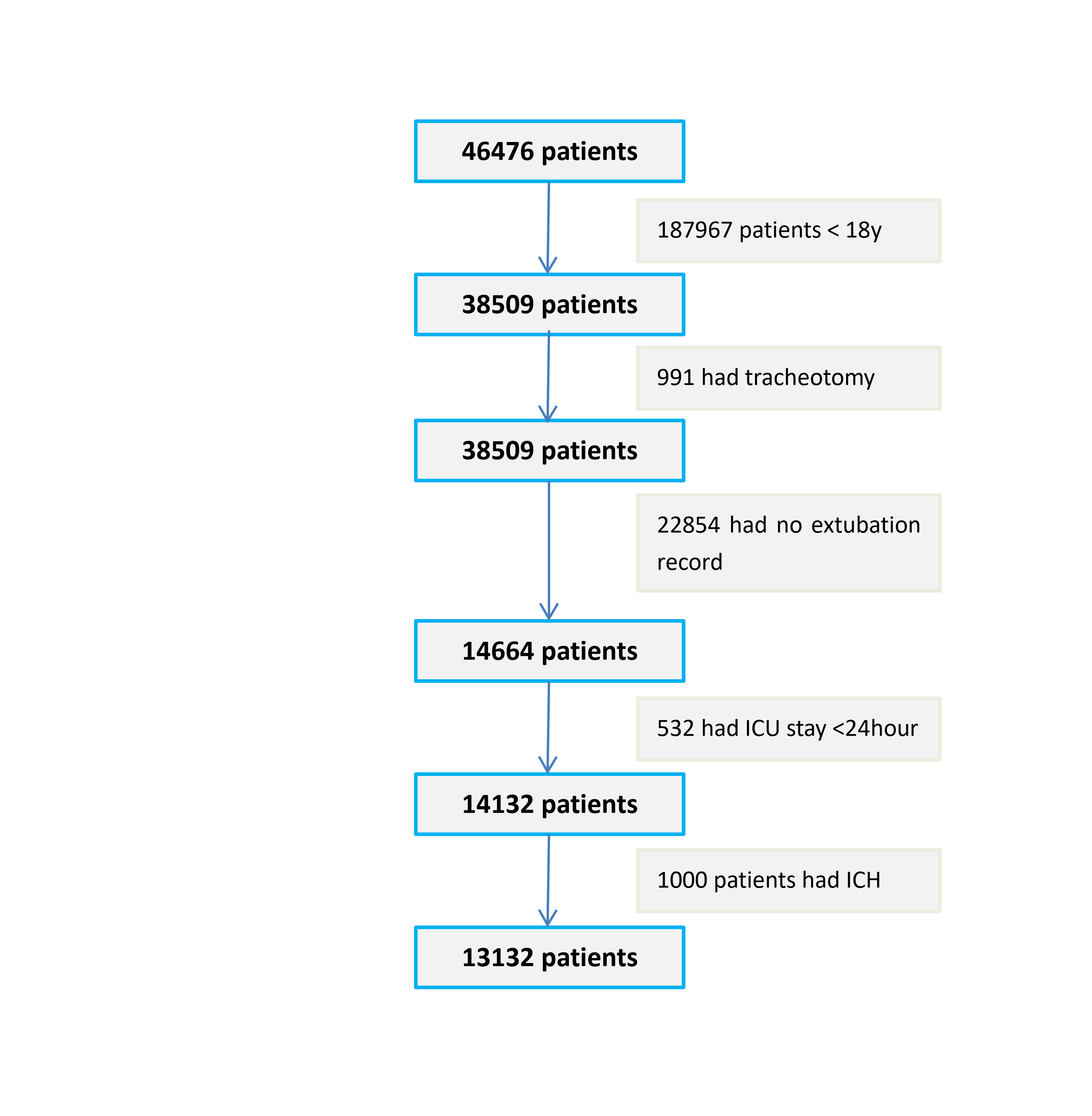


Figure S1 Flowchart of patient selection process

ICH, intracerebral haemorrhage


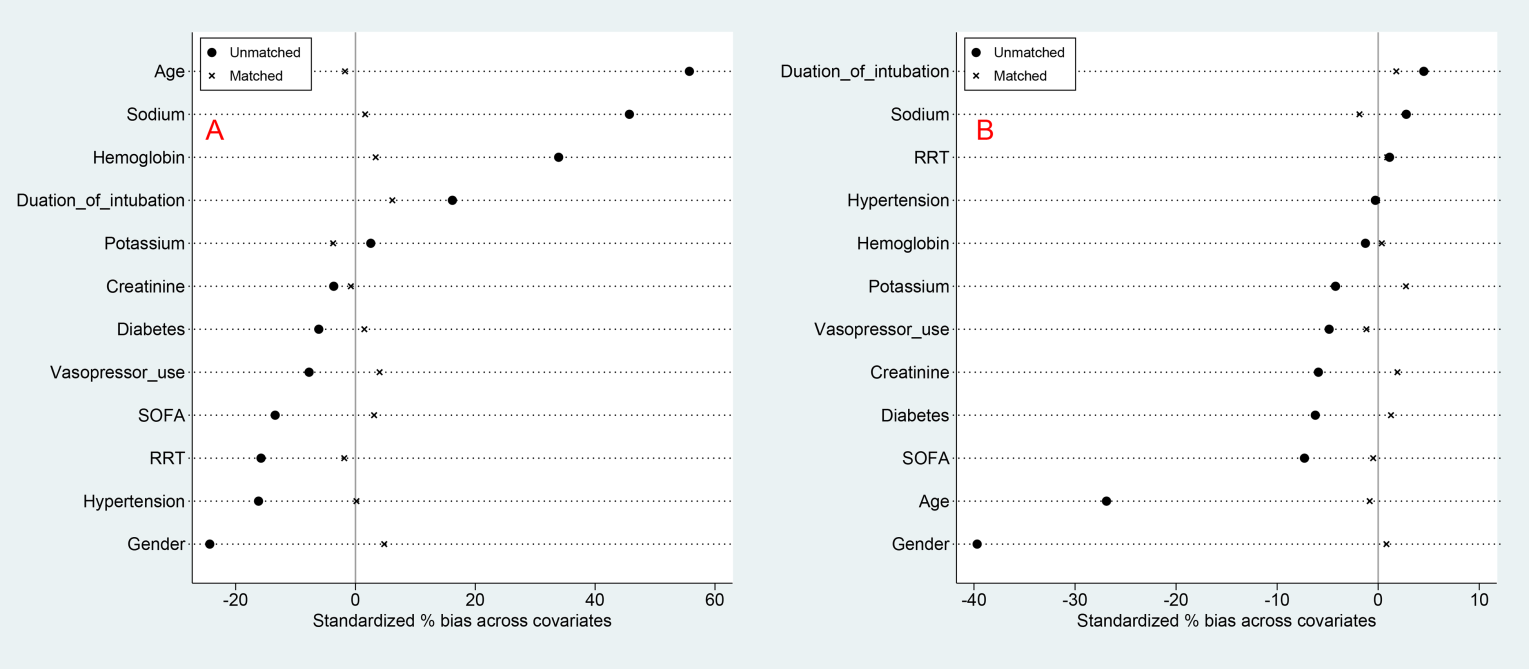


Figure S2 Overall quality of the matched samples after propensity score matching

Note: A COPD and Non-COPD patients; B Asthma and Non-asthma patients.

COPD, chronic obstructive pulmonary disease; SOFA, Sequential Organ Failure Assessment; RRT, renal replacement therapy

Table S1 Interaction between CRDs and post-extubation PH on re-intubation rate

|  | COPD vs. non-COPD  *P for interaction = 0.118* | | Asthma vs. non-asthma  *P for interaction = 0.075* | |
| --- | --- | --- | --- | --- |
|  | OR (95% CI) | P | OR (95% CI) | P |
| PH <7.35 | 4.0 (2.0 – 7.8) | < 0.001 | 0.9 (0.4 – 1.8) | 0.782 |
| PH 7.35 – 7.45 | 2.0 (1.2 – 3.2) | 0.003 | 0.9 (0.6 – 1.3) | 0.778 |
| PH >7.45 | 2.2 (1.3 – 3.6) | 0.002 | 1.3 (0.9 – 2.0) | 0.117 |

COPD, chronic obstructive pulmonary disease; CRDs, chronic respiratory diseases

|  | COPD vs. non-COPD  P for interaction = 0.787 | | Asthma vs. non-asthma  P for interaction = 0.109 | |
| --- | --- | --- | --- | --- |
|  | OR (95% CI) | P | OR (95% CI) | P |
| Sedation | 2.5 (1.6 – 3.9) | < 0.001 | 0.8 (0.6 – 1.2) | 0.512 |
| Without sedation | 2.6 (1.7 – 4.0) | < 0.001 | 1.4 (0.9 – 2.0) | 0.077 |

Table S2 Interaction between various CRDs and post-extubation sedation on re-intubation rate

COPD, chronic obstructive pulmonary disease; CRDs, chronic respiratory diseases

Table S3 Associations between clinical outcomes and different respiratory diseases

|  | CRDs | | | COPD | | | Asthma | | |
| --- | --- | --- | --- | --- | --- | --- | --- | --- | --- |
|  | Multivariable logistic model, aOR (95% CI)^a^ | p | Multivariable logistic model with bootstrapping, aOR (95% CI)^b^ | Multivariable logistic model, aOR (95% CI)^a^ | p | Multivariable logistic model with bootstrapping, aOR (95% CI)^b^ | Multivariable logistic model, aOR (95% CI)^a^ | p | Multivariable logistic model with bootstrapping, aOR (95% CI)^b^ |
| Re-intubation | 1.26 (1.04 – 1.53) | 0.020 | 1.26 (1.04 – 1.52) | 1.55 (1.14 – 2.09) | 0.004 | 1.56 (1.12 – 2.15) | 1.08 (0.83 – 1.40) | 0.532 | 1.08 (0.81 – 1.43) |
| In-hospital mortality | 0.93 (0.74 – 1.17) | 0.143 | 0.92 (0.74 – 1.18) | 1.32(1.05 – 1.63) | 0.010 | 1.33 (1.04 – 1.61) | 0.62 (0.42 – 0.92) | 0.018 | 0.61 (0.41 – 0.95) |

^Note: a Multivariable logistic model was used to evaluate the association between clinical outcomes and different respiratory diseases. All these models were adjusted for same co-variables, including age, hypertension, coronary disease, sepsis, renal replacement therapy, duration of intubation, haemoglobin level, white blood cell count, serum sodium level, and vasopressor use.^

^b 95% CI using bootstrap techniques (100 resamples) was adjusted for the same co-variables listed above.^

^Abbreviations: CRDs chronic respiratory diseases; COPD chronic obstructive pulmonary disease; aOR adjusted odds ratio.^
